# Vitamin D/VDR signaling inhibits LPS-induced IFNγ and IL-1β in Oral epithelia by regulating hypoxia-inducible factor-1α signaling pathway

**DOI:** 10.1186/s12964-019-0331-9

**Published:** 2019-02-27

**Authors:** Xuejun Ge, Lixiang Wang, Mengdi Li, Na Xu, Feiyan Yu, Fang Yang, Ran Li, Fang Zhang, Bin Zhao, Jie Du

**Affiliations:** 1 Department of Oral Medicine, Shanxi Medical University School and Hospital of Stomatology, NO. 56 Xinjian South Road, Taiyuan, 030001 Shanxi China; 2 Institute of Biomedical Research, Shanxi Medical University, Taiyuan, Shanxi China

**Keywords:** Vitamin D, Vitamin D receptor, HIF-1α, Cytokines, Oral lichen planus

## Abstract

**Background:**

Oral lichen planus (OLP) is known as a chronic inflammatory disease. Our recent studies have suggested that vitamin D/vitamin D receptor (VDR) signaling exerts its protective effects on oral keratinocyte apoptosis by regulating microRNA-802 and p53-upregulated modulator of apoptosis (PUMA), but its roles in oral epithelial inflammatory responses in OLP are still unknown. Herein, we identify lipopolysaccharide (LPS) is able to enhance interferon gamma (IFNγ) and interleukin-1 beta (IL-1β) productions in human oral keratinocytes (HOKs) dependent on hypoxia-inducible factor-1α (HIF-1α).

**Methods:**

HIF-1α and cytokines levels in HOKs were investigated by real-time PCR and western blotting after LPS challenge. The effects of 1,25(OH)_2_D_3_ on LPS-induced HIF-1α and cytokines were tested by real-time PCR, western blotting, siRNA-interference and plasmids transfection techniques. The roles of 1,25(OH)_2_D_3_ in regulating HIF-1α levels were investigated using western blotting, siRNA-interference, plasmids transfection and Chromatin Immunoprecipitation (ChIP) assays. Finally, HIF-1α, IFNγ and IL-1β expressions in oral epithelia derived from mice and individuals were measured by real-time PCR, western blotting and immunohistochemical staining.

**Results:**

As a critical regulator, vitamin D suppresses LPS-induced HIF-1α to block IFNγ and IL-1β productions. Mechanistically, vitamin D inactivates nuclear factor-κB (NF-κB) pathway and up-regulates von Hippel-Lindau (VHL) levels, leading to HIF-1α reduction. Moreover, HIF-1α status of oral epithelia is elevated in VDR^−/−^ mie as well as in VDR-deficient human biopsies, accompanied with increased IFNγ and IL-1β.

**Conclusion:**

Collectively, this study uncovers an unrecognized roles of vitamin D/VDR signaling in regulating cytokines in oral keratinocytes and reveals the molecular basis of it.

**Electronic supplementary material:**

The online version of this article (10.1186/s12964-019-0331-9) contains supplementary material, which is available to authorized users.

## Background

Oral lichen planus (OLP) is identified to be a chronic mucosal inflammatory condition [1]. The prevalence of this disorder is approximately 0.5–3% in adult and is expected to continue unabated in the coming decades [2]. OLP is considered as potentially malignant disorder and the malignant transformation rate is 0.4–5% as reported previously [2]. Although the pathogenesis remains largely a mystery, OLP is characterized by T cell-infiltrated band in lamina propria in oral mucosa [3]. In a broad consensus, a cause of disease progression in OLP is inflammatory response due to autoimmune reaction [4]. Furthermore, recently studies recapitulated that the invasion of bacteria of oral cavity through mucosal epithelium is also a contributor factor for inflammation in OLP, and interest in bacterial infection in the context of OLP continues to grow [5].

Hypoxia-inducible factor 1 (HIF-1), a member of the basic helix-loop-helix (bHLH) family, is activated in various kinds of cells under the inflammatory and hypoxic conditions [6, 7]. HIF-1 is known as a heterodimeric transcription factor, which is comprised of a regulated α subunit and a constitutively expressed β subunit [6]. In normoxia, HIF-1α subunits are hydroxylated by three prolyl hydroxylases (PHD1–3) on proline residues, and then targeted by the von Hippel-Lindau (VHL) for subsequent proteasomal degradation [8]. Under hypoxia condition, HIF-1α cannot be hydroxylated by PHDs, leading to its stabilization [8, 9]. Furthermore, α subunit is able to be induced in inflammatory microenvironment in an oxygen-independent manner [6]. It is reported that Lipopolysaccharide (LPS) is able to promote HIF-1α status by increasing succinate levels in macrophages and activating nuclear factor-κB (NF-κB) pathway in myeloid cells [10, 11], and conserved κB sites are located in the promoters of human and mouse HIF-1α genes [12]. In turn, the induced HIF-1α is claimed to enhance transcription of cytokines such as interleukin-1 beta (IL-1β) and interferon gamma (IFNγ) in immune cells, accelerating inflammatory response [13, 14].

Vitamin D is a pleiotropic hormone with a broad range of biological activities and its active form is 1,25-dihydroxyvitamin D (1,25(OH)_2_D_3_) [15]. 1,25(OH)_2_D_3_ exerts its biological functions dependent on the vitamin D receptor (VDR), which is a member of nuclear hormone receptor superfamily and markedly expressed in a wide variety of cells [16]. While vitamin D/VDR signaling is known for its effect on bone homeostasis, its suppressive regulation concerning inflammatory response is becoming more attractive [17, 18]. Some studies have confirmed that vitamin D/VDR signaling suppresses the activation of NF-κB pathway in embryonic fibroblasts and intestinal epithelial cells [19, 20]. Furthermore, vitamin D is reported to suppress HIF-1α expression in osteoclasts [21] and tumor necrosis factor alpha (TNFα) plays a role in downregulating VDR level [22]. Although vitamin D/VDR signaling has been confirmed to play a protective role in the progression of OLP by mediating cytokines [23], the molecular underpinnings have yet to be clearly explained. To better understand it, we set out to explore the mechanism by which vitamin D/VDR signaling regulates inflammation in oral keratinocytes. In this study, we demonstrate that vitamin D/VDR signaling suppresses IFNγ and IL-1β productions by regulating LPS-induced HIF-1α and offer a unique window into the understanding of OLP pathogenesis.

## Methods and materials

### Cell culture

The cell line, human oral keratinocyte (HOK), was got from Chinese Beijing North Institution and cultured at 37 °C and 5% CO_2_ with oral keratinocyte medium (ScienCell Research Laboratories). Cells were stimulated with 100 ng/ml LPS (Sigma, MO) for 24 h after 12-h pre-treatment of 20 nM 1,25(OH)_2_D_3_ (Yanchun Li lab, University of Chicago) or 20 nM BAY 11–7082 (Biyuntian, China). In separate experiments, VDR, IκB Kinase β (IKKβ) and HIF-1α plasmids (4 μg) were transfected using Lipofectamine 2000 (Invitrogen, Grand Island, NY), following 12-h LPS (100 ng/ml) or 1,25(OH)_2_D_3_ (20 nM) pre-treatment. Scramble or human specific siRNA (40 μM, GenePharma, China) was transfected by Lipofectamine 2000 (Invitrogen, NY) as well for HIF-1α or VHL silencing. The target sequences of hHIF-1α-siRNA and hVHL-siRNA were 5′-AGAGGUGGAUAUGUGUGGGdTdT-3′ and 5′ -GGACACACGATGGGCTTCTGGTTAAC-3′, respectively [24, 25]. VDR and IKKβ plasmids were obtained from Yanchun Li lab (University of Chicago), HIF-1α plasmid was got from Juanjuan Xiang (Central South University, China). All the HOKs used in this study were the third passage when cells became stable. In addition, cell viability and proliferation rates were not affected by any of the treatments.

### Animal studies

VDR^−/−^ and VDR^+/+^ C57BL/6 male mice (6–8-week old) were used in this study. VDR^−/−^ mice were generated in term of the approach described previously [26]. 2 mm tails of mice were obtained and DNA was isolated for genotyping. To deplete bacteria in oral cavity, mice were subjected to an antibiotic cocktail (Sigma) in drinking water (1300 mg/l of metronidazole and 660 mg/l of levofloxacin) for 2 weeks, and then 1-day treatment without antibiotics according to previous studies [27]. After sacrifice, buccal epithelial tissues were got for RNA or protein isolation. All animal studies were approved by the Institutional Ethical Committee of Shanxi Medical University.

### Human biopsies

Snap-frozen human buccal mucosal samples were obtained from patients suffered with OLP at the Stomatological Hospital of Shanxi Medical University as described previously [23]. Patient inclusion criteria and identification of OLP were established based on the WHO diagnostic criteria and published investigations [28, 29]. Healthy control tissues were got from retained wisdom teeth extraction operations without any inflammation and other kinds of diseases. Informed consent including signature was collected from each participant. This study was approved by the Institutional Ethical Committee of Shanxi Medical University. The size calculations of samples were all 3 mm × 3 mm or so. OLP patients denied other local (e.g. periodontitis) or systemic diseases, and they did not receive any treatments by the sampling time. Details of patients were provided before [23, 30].

### Isolation of mucosal epithelium

Oral mucosal samples derived from both human and mice were washed using cold phosphate buffer solution (PBS), and then digested at 4 °C using 0.25% dispase II (StemCell Technologies, Canada) for 12 h, followed by epithelial layer separation. Muscle forceps were used to separate epithelium and lamina propria layers. The detached epithelium was cut into small pieces and placed into 0.05% Trypsin-0.53 mM EDTA (Gibco) for 10-min incubation at 37 °C. Suspended single cells were centrifuged and pellet was got for protein or RNA isolation [31].

### Western blot

HOK cells and mucosal epithelia were denatured in laemmli buffer, centrifuged and then heated for 10 min at 95 °C. Proteins were separated by SDS-PAGE, and then electroblotted onto PVDF membranes (Millipore). Western blot analyses were carried out as described [32]. The following primary antibodies (1:1000 dilution) were used in this study: anti-IKKβ, anti-phospho-NF-κB p65, anti-NF-κB p65 from Cell Signaling, anti-Toll-like receptor 4 (TLR4), anti-IFNγ, anti-IL-1β, anti-TNFα, anti-interleukin 6 (IL-6), anti-VDR and anti-β-actin from Santa Cruz Biotechnology, anti-HIF-1α from Millipore, anti-PHD1, anti PHD2, and anti-VHL from Abcam.

### Reverse transcription-quantitative PCR (RT-qPCR)

Total RNAs of HOKs and oral epithelia were extracted with TRIzol reagent (Invitrogen). First-strand cDNA templates were synthesized using PrimeScript RT reagent kit (TaKaRa) in the light of manufacturer’s instructions. Quantitative PCR was conducted using SYBR Premix Ex kit (TaKaRa) according to manufacturer’s instructions. Relative amount of transcripts was calculated according to the 2^-ΔΔCt^ formula. Sequences of PCR primers were provided in Table 1.

**Table 1:** Primer sequences involved in this work

Primer name	Forward(5′-3′)	Reverse(5′-3′)
hHIF-1α	GAACGTCGAAAAGAAAAGTCTCG	CCTTATCAAGATGCGAACTCACA
hTNFα	CGAGTGACAAGCCTGTAGC	GGTGTGGGTGAG-GAGCACAT
hIL-1β	GCTGAGGAAGATGCTGGTTC	GTGATCGTACAGGTCGCATCG
hIFNγ	TGAACATGATGGATCGTTGG	CATTCACTTTGCTGGCAGTG
hIL-6	AAATGCCAGCCT-GCTGACGAAC	AACAACAATCTGAGGTGCCCATGCTAC
hTREM-1	GGCAGATAATAAGGGACGGAGAG	CATTCGGACGCGCAGTAAA
hGADPH	ACCACAGTCCATGCCATCAC	TCCACCACCCTGTTGCTGTA
mIL-1β	CAGGATGAGGACATGAGCACC	CTCTGCAGACTCAAACTCCAC
mIFNγ	CGGCACAGTCATTGAAAGCCTA	GTTGCTGATGGCCTGATTGTC
mGADPH	TGTGTCCGTCGTGGATCTGA	CCTGCTTCACCACCTTCTTGA
HIF-1α κB ChIP	GACAAGCCACCTGAGGAGAG	CACGCGGAGAAGAGAAGGAA

### Chromatin immunoprecipitation (ChIP) assays

ChIP assays were carried out as described before [33]. Briefly, HOK cells were washed with cold PBS, crosslinked with 1% formaldehyde and stopped by 0.125 M glycine. Then, cells were sonicated in ChIP lysis buffer to generate 600 bp fragments, followed by an overnight incubation with anti-NF-κB antibody at 4 °C. Real-time PCR was performed using human NF-κB primer (Table 1).

### Immunostaining

Freshly harvested human oral mucosal tissues were fixed overnight in 10% formalin. For immunostaining assay, sections (4 μm) were treated overnight with VDR or HIF-1α antibodies (1: 100 dilution), followed by secondary antibodies (1: 200 dilution) and diaminobenzidine (DAB) incubation. Slides were observed by a fluorescence microscope system.

### Statistical analysis

Data values were presented as means ± SD. Statistical comparisons were implemented using unpaired two-tailed Student’s *t*-test or one-way analysis of variance (ANOVA) as appropriate, with *P* < 0.05 being considered statistically significant.

## Results

### LPS up-regulates HIF-1α and cytokines levels in HOKs

**Fig. 1:**
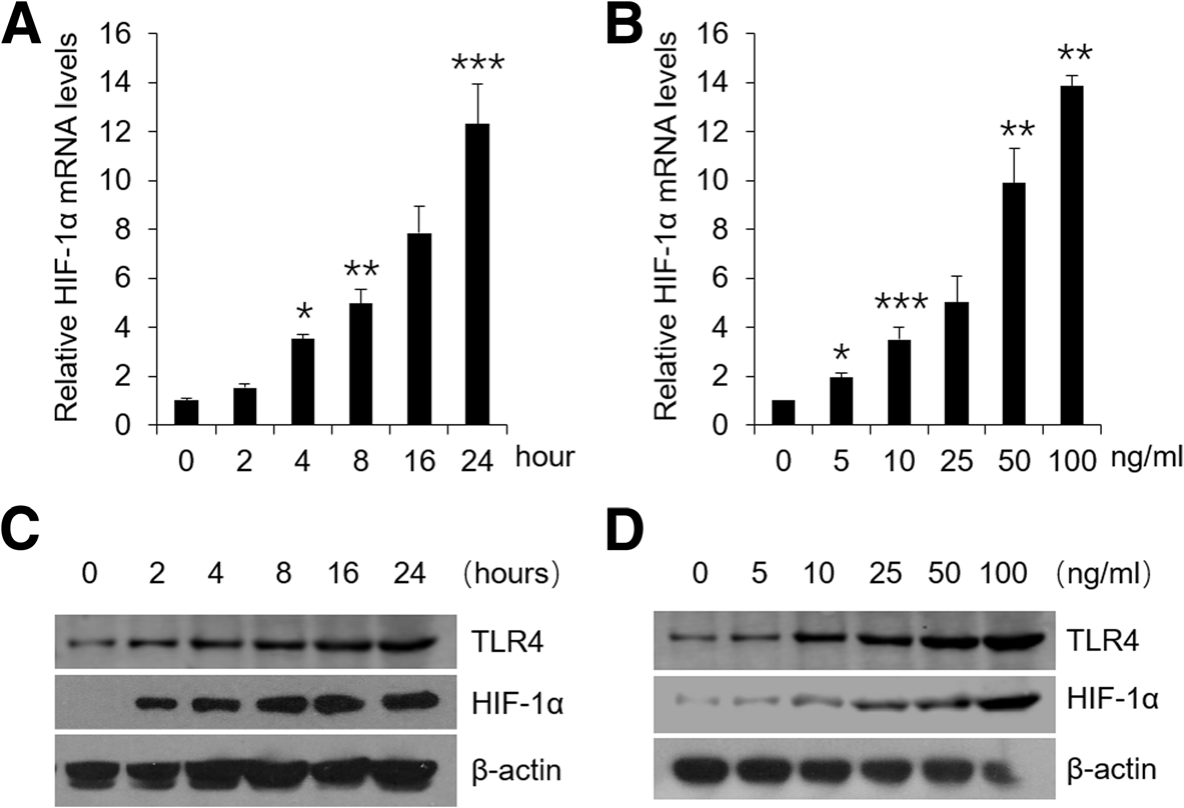
LPS improves HIF-1α levels in HOKs. **a** Real-time PCR quantification of HIF-1α in HOKs stimulated with time course-dependent LPS (100 ng/ml) or saline. **b** Real-time PCR quantification of HIF-1α in HOKs treated with concentration-dependent LPS or saline for 24 h. **c** Western blot analyses of HOK lysates stimulated with time course-dependent LPS (100 ng/ml) or saline. **d** Western blot analyses of HOK lysates treated with concentration-dependent LPS or saline for 24 h. *P < 0.05, **P < 0.01, ***P < 0.001 vs. corresponding controls. All assays were conducted for three times, *n* = 3

HIF-1α can be induced by LPS in immune cells as reported before [10, 11]. To confirm it in oral keratinocytes, we carried out in vitro assays in HOKs. As shown in Fig. 1, HIF-1α mRNA expression was increased by LPS treatments in a time- and dose-dependent manner (Fig. 1, a and b). In agreement, with a concomitant increase of TLR4, LPS also promoted HIF-1α protein levels (Fig. 1c and d). Are the levels of cytokines augmented as well under LPS challenge in HOKs? To address it, we tested four key cytokines (IFNγ, IL-1β, TNFα and IL-6) in the following experiments. As expected, qPCR assays showed that mRNA levels of these cytokines were elevated (Additional file 1: Figure S1, A and B) and Elisa data demonstrated enhanced protein productions (Additional file 1: Figure S1, C and D).

### 1,25(OH)_2_D_3_ exerts its suppressive effects on LPS-induced HIF-1α and cytokines

**Fig. 2:**
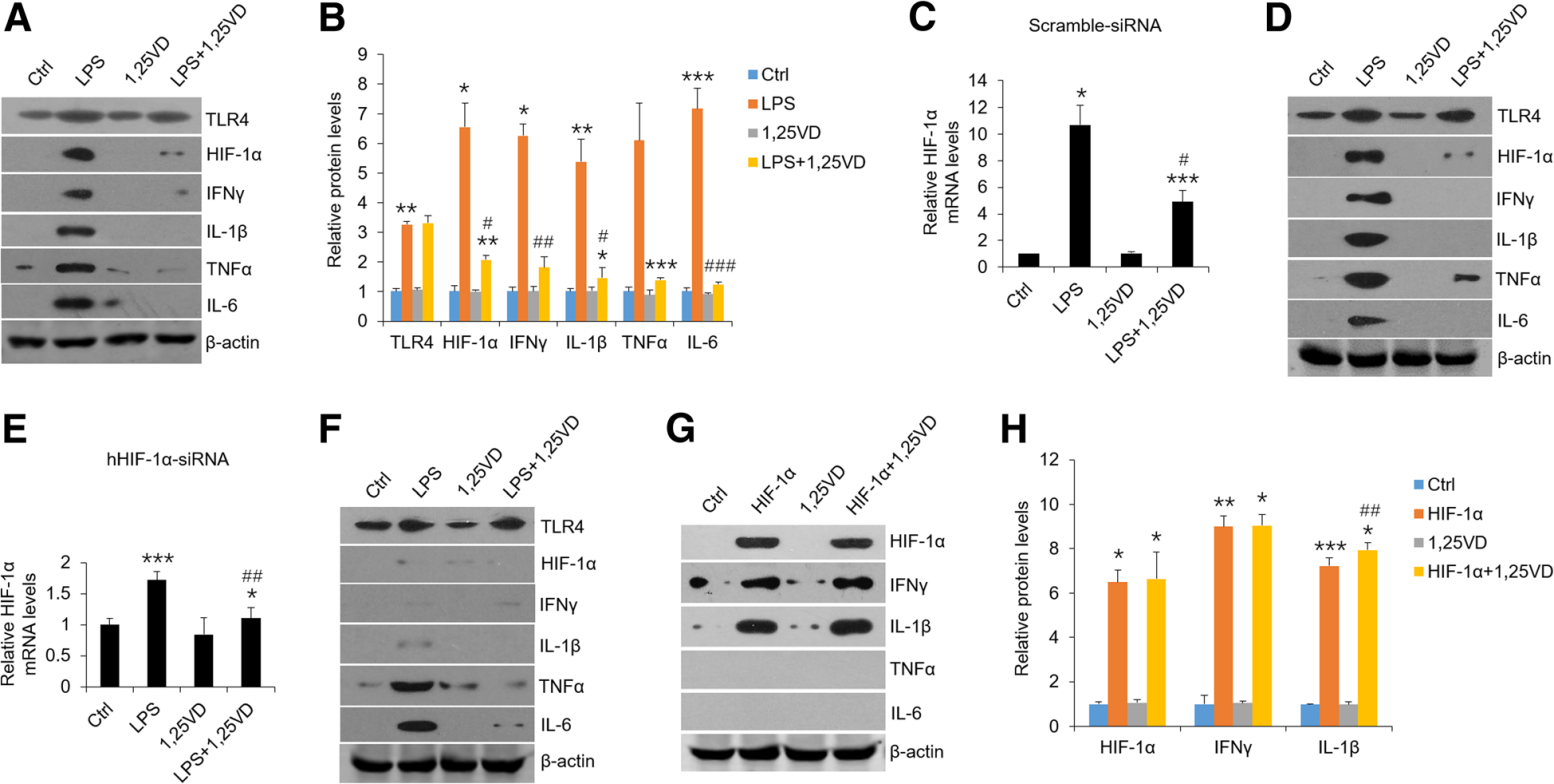
1,25(OH)_2_D_3_ regulates IFNγ and IL-1β expressions dependent on HIF-1α pathway. **a** and **b** Western blot measurements of HOKs treated with LPS (100 ng/ml) or saline in the presence or absence of 20 nM 1,25(OH)_2_D_3_ (**a**), and corresponding densitometric analysis (**b**). **c** and **d** Real-time PCR quantification (**c**) and western blot analyses (**d**) of scramble siRNA-transfected HOKs followed by different treatments as shown. **e** and **f** Real-time PCR quantification (**e**) and western blot analyses (**f**) of hHIF-1α-siRNA-transfected HOKs followed by various stimulations as indicated. **g** and **h** Western blot measurements of HOKs transfected with HIF-1α plasmids or control vector with or without 20 nM 1,25(OH)_2_D_3_ pre-treatment (**g**), and densitometric analysis (**h**). *P < 0.05, **P < 0.01, ***P < 0.001 vs. corresponding controls; #*P* < 0.05, ##*P* < 0.01, ###*P* < 0.001 vs. LPS or HIF-1α; Ctrl, control; 1,25VD, 1,25(OH)2D3. All assays were conducted for three times, *n* = 3

Vitamin D has been reported to block immune response by virtue of regulating T cell differentiation and relieving cytokines secretion [17]. Here, we examined vitamin D’s roles in HOKs in the presence of LPS. As shown in Fig. 2, 1,25(OH)_2_D_3_ markedly reversed LPS-induced HIF-1α and cytokines expressions (Fig. 2a and b), suggesting vitamin D plays an inhibitory role in inflammatory response in HOKs. As previous studies stated that HIF-1α induces the transcripts of IFNγ and IL-1β in macrophage [13, 14], we then exploited the relationships among vitamin D, HIF-1α and cytokines in oral keratinocytes. In comparison with scramble control, we found that LPS lost its ability to induce IFNγ and IL-1β in HOKs which were transfected with hHIF-1α-siRNA, while vitamin D suppressed all the four cytokines in both groups regardless of HIF-1α silencing (Fig. 2c-f). Chetomin, which prevents the formation of the transcriptionally competent HIF-1 complex, inhibited LPS-induced HIF-1α and downstream cytokines productions as well (Additional file 2: Figure S2A). Consistently, HIF-1α overexpression induced IFNγ and IL-1β but not TNFα and IL-6 in HOKs, and vitamin D failed to reverse it (Fig. 2g and h). These findings indicate that vitamin D regulates HIF-1α expression to suppress IFNγ and IL-1β productions in HOKs with LPS treatment.

### 1,25(OH)_2_D_3_ inhibits HIF-1α levels by regulating NF-κB pathway as well as by inducing VHL expression

**Fig. 3:**
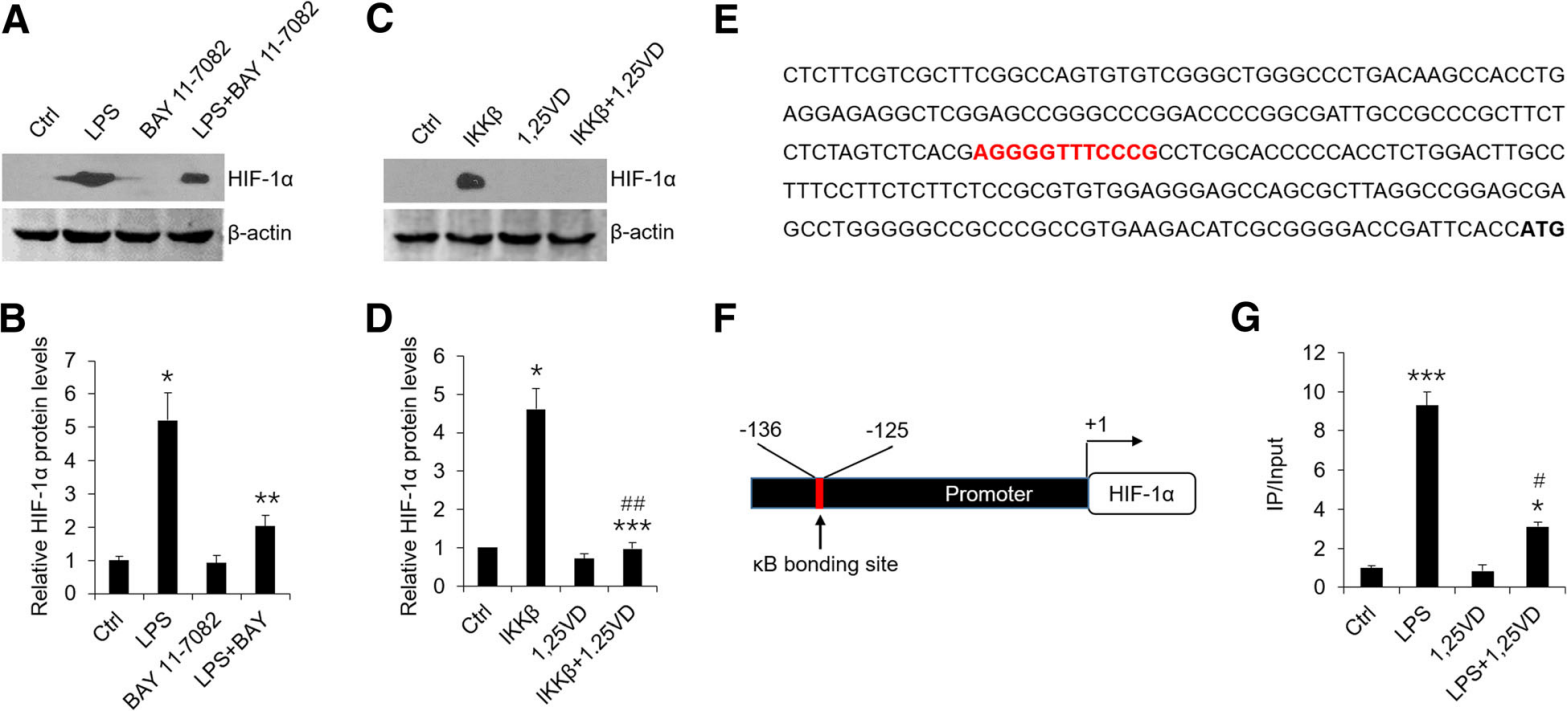
1,25(OH)_2_D_3_ mediates HIF-1α expression via NF-κB pathway. **a** and **b** Western blot analyses (**a**) of HOKs challenged with LPS or saline in the presence or absence of BAY 11–7082 (20 nM), and densitometric analysis (**b**). **c** and **d** Western blot analyses (**c**) of HOKs transfected with IKKβ plasmids or empty vector in the presence or absence of 1,25(OH)_2_D_3_, and densitometric analysis (**d**). **e** A predicted κB binding site (red) in human HIF-1α gene promoter. The initiation codon was labeled in bold font. **f** NF-κB binding site harbored in HIF-1α gene promoter was located between − 125 and − 136. **g** ChIP assays of HOKs treated by LPS or saline in the presence or absence of 1,25(OH)_2_D_3_. *P < 0.05, **P < 0.01, ***P < 0.001 vs. corresponding controls; #*P* < 0.05, ##*P* < 0.01 vs. LPS or Ikkβ; Ctrl, control; 1,25VD, 1,25(OH)2D3. All assays were conducted for three times, *n* = 3

Some observations have suggested that there are κB bonding sites in the promoter of HIF-1α gene [12]. To address whether LPS induces HIF-1α via NF-κB pathway in HOKs, we pre-treated cells with BAY 11–7082 and IMD-0354 which are known to block NF-κB signaling pathway. As shown, LPS-induced high levels of HIF-1α were reduced partially in the presence of BAY 11–7082 (Fig. 3a and b) or IMD-0354 (Additional file 2: Figure S2B). On the contrary, in the gain-of-function assays HIF-1α overexpression was found following IKKβ transfection (Fig. 3c and d), suggesting activated NF-κB pathway induces HIF-1α production. In particular, vitamin D blocked IKKβ-induced HIF-1α expression considerably (Fig. 3c and d), which implies vitamin D suppresses HIF-1α in a NF-κB-dependent fashion. To dissect the molecular underpinning of this regulation, we carried out bioinformatics analysis of the promoter region of HIF-1α gene using JASPAR CORE 2016 database and identified a potential NF-κB binding site (Fig. 3e). We then designed primers flanking this binding site for ChIP assays (Fig. 3f). Consistently, NF-κB p65 bound to κB site strongly after LPS treatment while vitamin D disrupted this binding process largely (Fig. 3g). These results decipher the mechanism by which vitamin D mediates HIF-1α mRNA expression.

**Fig. 4:**
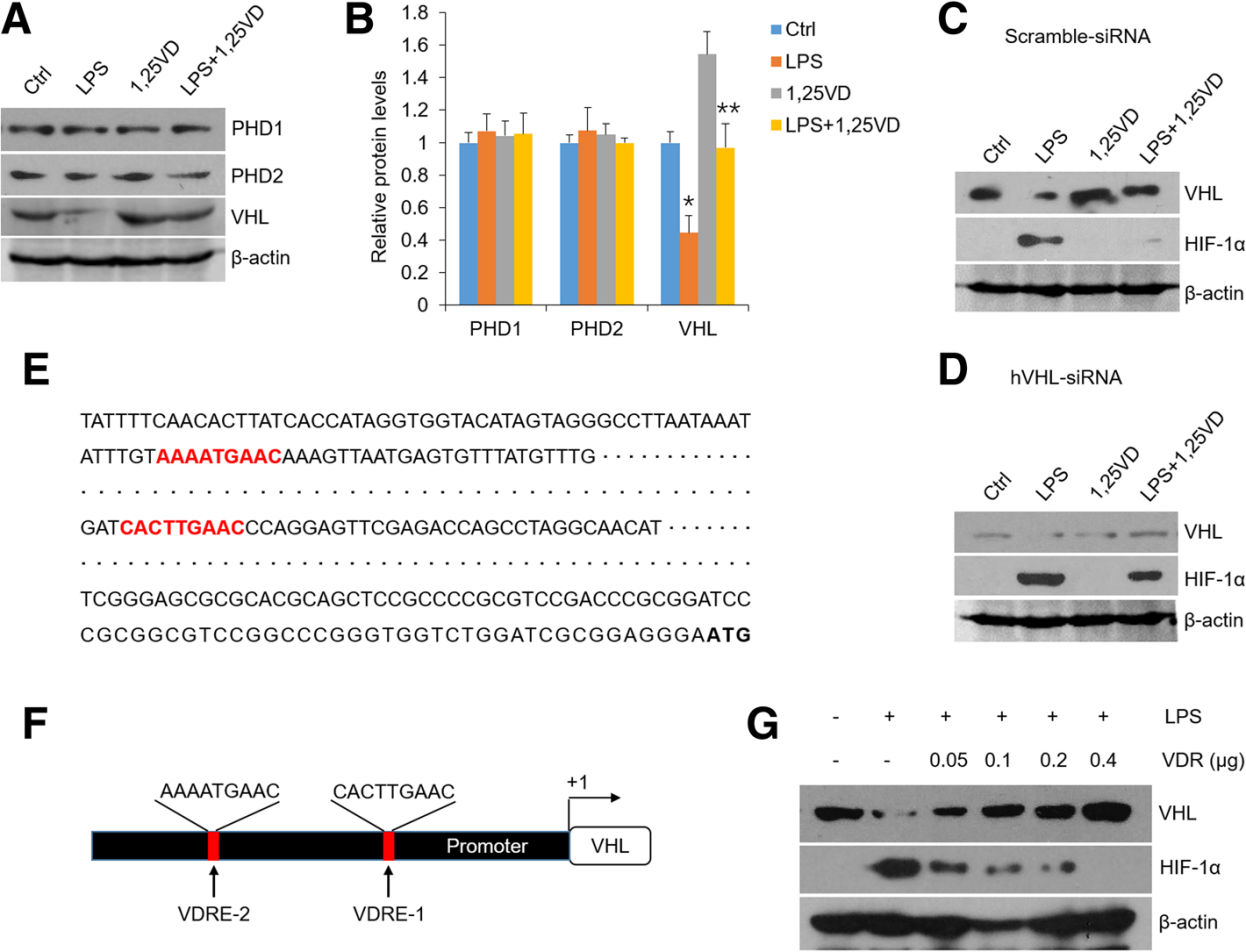
1,25(OH)_2_D_3_ induces VHL transcripts to regulate HIF-1α levels. **a** and **b** Western blot analyses (**a**) of HOKs challenged with LPS or saline following 1,25(OH)_2_D_3_ treatments, and densitometric analysis (**b**). **c** and **d** western blot analyses of scramble-siRNA- (**c**) or hHIF-1α-siRNA-transfected (**d**) HOKs followed by various stimulations as indicated. **e** Two putative VDR binding sites (red) in human VHL gene promoter. The initiation codon was labeled in bold font. **f** Promoter of VHL gene harboring 2 VDR binding sites. VDRE1 was located between − 505 and − 512, and VDRE2 was located between − 849 and − 857. **g** Western blot analyses of HOKs transfected with various concentrations of VDR plasmid followed by LPS or saline challenge as indicated. *P < 0.05, **P < 0.01 vs. corresponding controls; Ctrl, control; 1,25VD, 1,25(OH)2D3. All assays were conducted for three times, *n* = 3

Since PHDs and VHL play crucial roles in the degradation of HIF-1α protein [6], we further tested these degradation-related factors following LPS and vitamin D treatments. As shown in Fig. 4, LPS down-regulated VHL expression but not PHD1 and PHD2, while 1,25(OH)_2_D_3_ stopped VHL reduction (Fig. 4a and b). Importantly, 1,25(OH)_2_D_3_ failed to inhibit HIF-1α increase remarkably in hVHL-siRNA group compared with scramble control, providing a proof that vitamin D regulates HIF-1α by increasing VHL expression. To address the mechanism whereby vitamin D mediates VHL, sequence analysis regarding VHL gene promoter region was performed and two potential VDR elements were identified (Fig. 4e and f), suggesting VDR may act as a transcription factor to induce VHL transcripts. The evidence to support this hypothesis is that VHL levels were increased robustly when increasing amounts of VDR were expressed in HOKs (Fig. 4g). These results uncover the mechanism whereby vitamin D mediates HIF-1α protein expression.

### HIF-1α, IFNγ and IL-1β are highly induced in oral epithelia derived from VDR^−/−^ mice and OLP patients

**Fig. 5:**
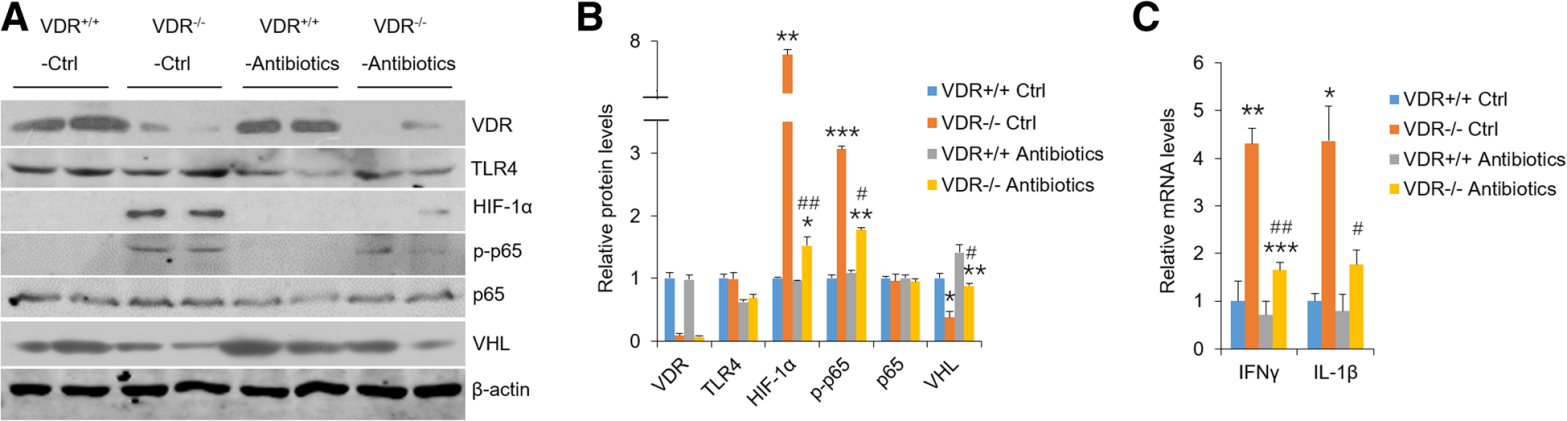
VDR depletion promotes HIF-1α levels of mouse oral epithelium in the presence of bacteria. **a** and **b** Western blot analyses (**a**) of oral epithelia lysates in the 4 groups of mice as indicated, and densitometric quantitation of these data (**b**). **c** Real-time PCR analyses of transcript levels of IFNγ and IL-1β in mouse oral epithelium. *P < 0.05, **P < 0.01, ***P < 0.001 vs. VDR^+/+^ controls; #*P* < 0.05, ##*P* < 0.01 vs. VDR^−/−^ controls; *n* = 5. Ctrl, control

**Fig. 6:**
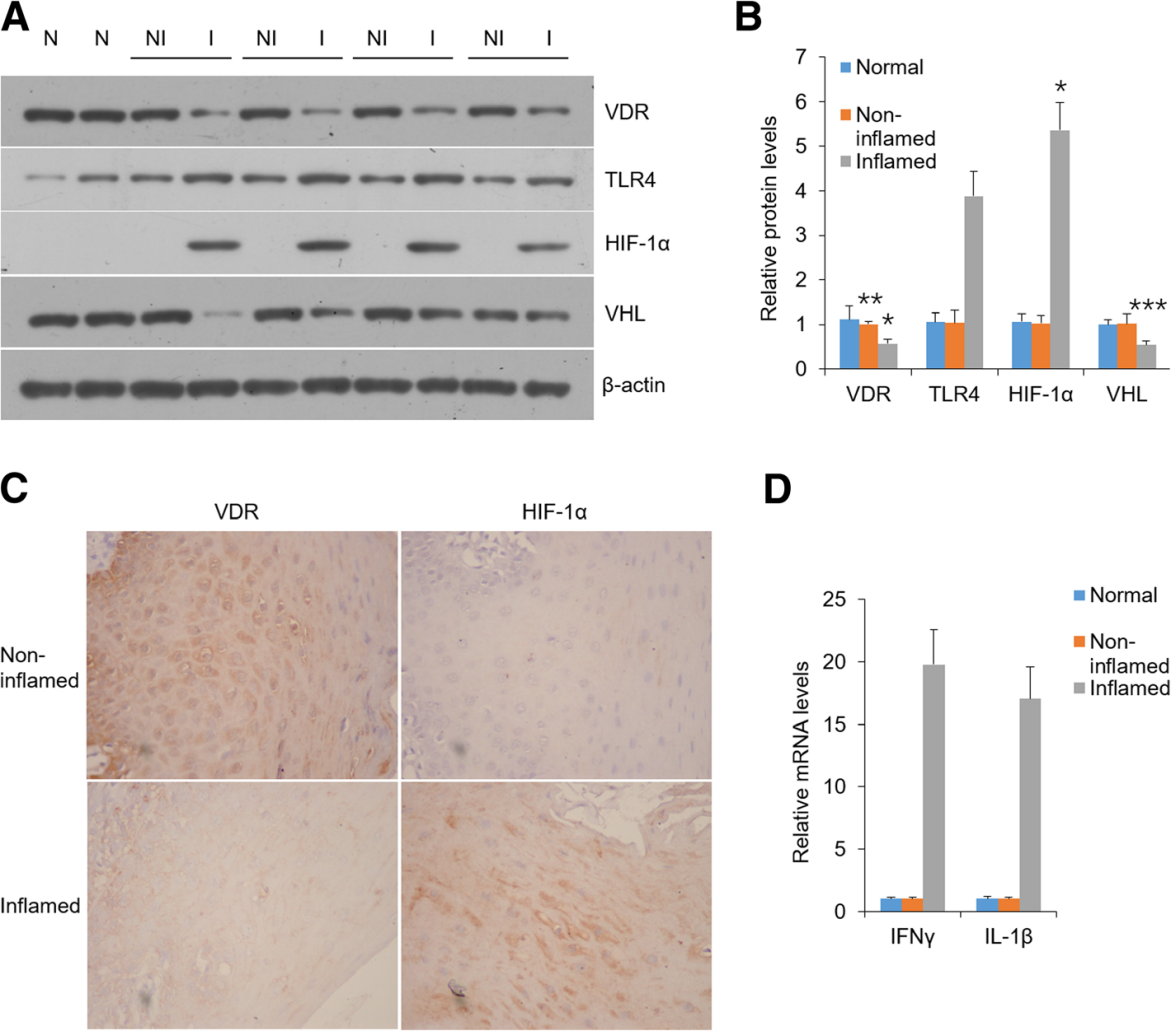
VDR decrease is associated with HIF-1α up-regulation in human oral biopsies. **a** and **b** Western blot analyses (**a**) of human oral epithelia lysates with antibodies as indicated, and densitometric quantitation of these data (**b**), N = normal; NI = non-inflamed; I = inflamed. **c** Immunostaining of sections of human oral samples. **d** Real-time PCR analyses of transcript levels of IFNγ and IL-1β in human oral epithelium. *P < 0.05, **P < 0.01, ***P < 0.001 vs. corresponding normal. Normal, healthy control; Non-inflamed, non-diseased tissue of OLP patients; Inflamed, diseased tissues of OLP patients. Normal, *n* = 7; OLP patients: *n* = 28

To explore the role of VDR in HIF-1α expression in vivo, we tested oral epitheliums which were isolated from VDR^−/−^ and VDR^+/+^ mice. Western blot data showed VDR deletion in control mice with normal microflora up-regulated HIF-1α and phosphor-p65 expressions and down-regulated VHL levels compared with VDR^+/+^ control mice, suggesting VDR is required to maintain normal status of HIF-1α and cytokines (Fig. 5a-b). In comparison, VDR^−/−^ mice subject to antibiotics exhibited reduced HIF-1α and phosphor-p65 as well as up-regulated VHL (Fig. 5a and b). Accordantly, accompanied with VDR and VHL decrease, oral inflamed samples from OLP patients showed high levels of HIF-1α and TLR4 (Fig. 6a-c). Triggering receptor expressed on myeloid cells type-1 (TREM-1), which is reported to induce HIF-1α with LPS treatment [34], was also enhanced considerably in OLP inflamed biopsies (Additional file 2: Figure S2C). What is more, transcripts of IFNγ and IL-1β were robustly promoted in VDR^−/−^ mice and human lesion biopsies (Figs. 5c and 6d), consistent with the results in vitro. These observations support the notion that VDR lack in oral epithelia augments HIF-1α levels and worsens inflammatory response, which is dependent on bacteria stimulation.

## Discussion

OLP is a helper T-cell type 1-dominated inflammatory disease and featured with massive sub-epithelial lymphocytic infiltration, a large number of intra-epithelial lymphocytes and destruction of basal keratinocytes [4]. Although progress is being made, the pathogenesis of OLP is less well understood. Previously studies mainly paid more attentions to immune reactions of lamina propria and apoptosis of epithelium [35–37], but little is known about the epithelial inflammation in OLP. Here, we have identified Vitamin D/VDR as a critical regulator in LPS-induced inflammatory response in oral keratinocytes. Previous studies reported that LPS induces IFNγ and other cytokines productions in oral epithelial cells [38, 39]. Consistently, our in vitro data support the notion that LPS induces HIF-1α and cytokines expressions in HOKs. Importantly, our western blot data show that vitamin D plays a suppressive role in HIF-1α as well as cytokines in HOKs, demonstrating its novel function on OLP. To explore the molecular mechanism of vitamin D’s action, we transfected siRNA and HIF-1α plasmids into HOKs and found that vitamin D inhibits IFNγ and IL-1β expressions in a HIF-1α-dependent way. These findings are in agreement with the point that IFNγ and IL-1β binding sites are harbored in the promoter region of HIF-1α gene [13, 14]. Interestingly, our results manifest that LPS-induced enhanced levels of TNFα and IL-6, which are suppressed by vitamin D, appear to be not regulated by HIF-1α. The reason underling the discrepancy should be that TNFα and IL-6 are mediated by NF-κB signaling pathway directly as described previously [23].

**Fig. 7:**
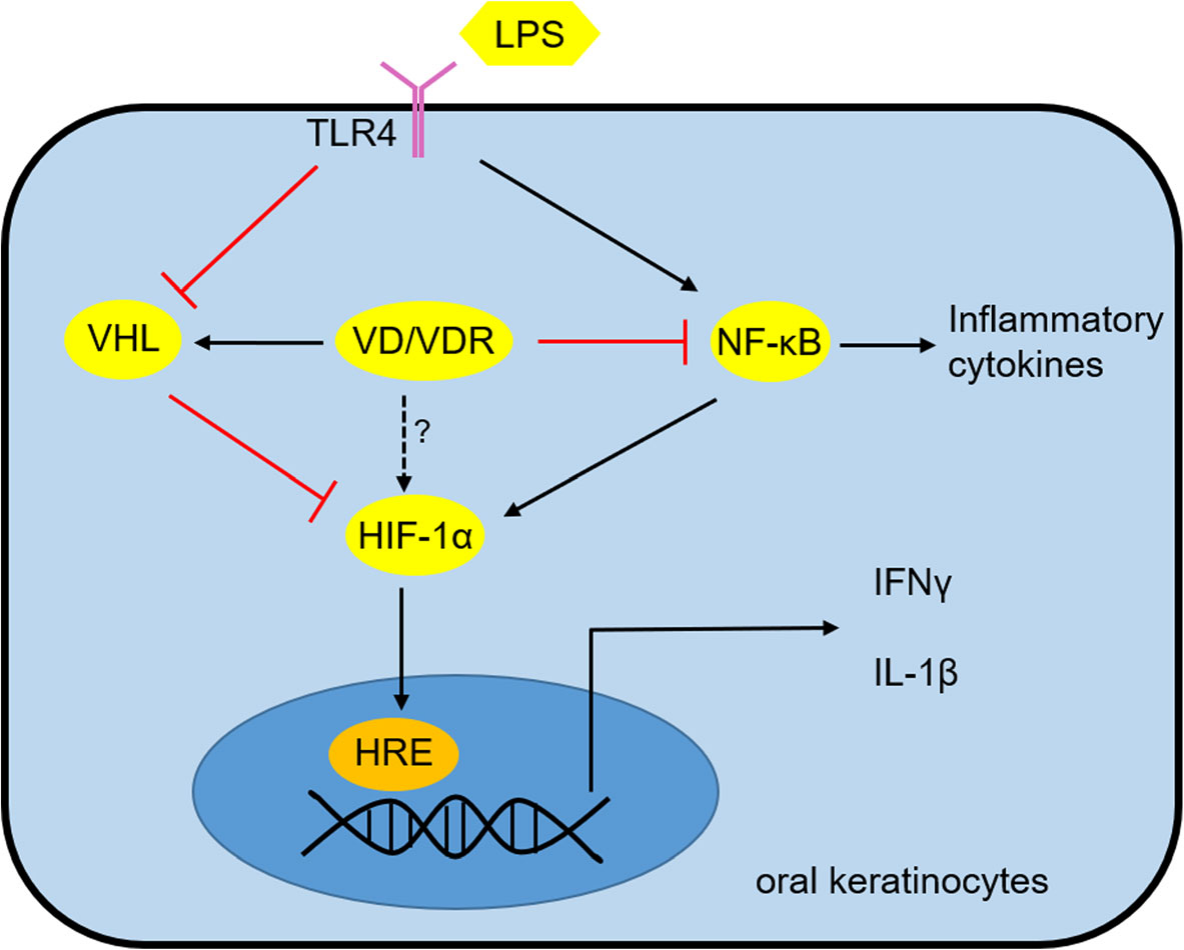
Schematic illustration of vitamin D/VDR’s regulation on HIF-1α as well as IFNγ and IL-1β in oral keratinocytes

HIF-1α is very hard to be detected in normal condition, but highly expressed in hypoxic or inflammatory situation in a great variety of cells [6]. HIF-1α activity in the epithelium is also important in O_2_ sensing and epithelial innate immunity [40]. Many studies have demonstrated that it can be induced by activated NF-κB pathway [12, 41]. Consistently, our loss-of-function and gain-of-function data support a regulatory role of NF-κB pathway in HIF-1α expression in oral epithelial cells. Previous studies have revealed that vitamin D blocks NF-κB activity through promoting the interaction between VDR and IKKβ [20]. In this study, we identified a putative κB bonding site in the promoter region of HIF-1α gene and found that 1,25(OH)2D3 disrupts LPS-induced p65 binding to the element of HIF-1α gene, resulting in the reduction of HIF-1α expression. 1,25(OH)_2_D_3_ has been stated to upregulates HIF-1α via mTOR signaling in human monocytes and breast epithelial cells [42, 43], while other studies uncovered that HIF-1α status is attenuated by 1,25(OH)_2_D_3_ treatment in human cancer cells [44]. This discrepancy may result from different types of cell lines used in these studies. In this investigation, we have confirmed that vitamin D reverses LPS-induced overexpression of HIF-1α in HOKs by impeding NF-κB pathway (Fig. 7).

PHDs and VHL are crucial factors which are required for HIF-1α proteasomal degradation [6]. In the following section, we found LPS exerts suppressive effects on VHL expression rather than PHD1 and PHD2 (PHD3 is not detectable) in HOKs, which is consistent with the notion that LPS down-regulates VHL expression in human hepatic stellate cells [45]. These results note that VHL likely plays a protective role in inflammatory diseases, in agreement with previous studies concerning hepatic fibrosis [46]. Furthermore, we have confirmed that vitamin D reverses VHL expression in HOKs with LPS challenge, and siRNA interference data show that vitamin D dose regulate HIF-1α dependent in part on VHL. The new finding above raises a crucial question regarding the molecular basis of vitamin D’s mediation on VHL. We then identified two VDR elements in the promoter region of VHL gene, and proved that overexpression of VDR increases VHL and decreases HIF-1α using plasmids transfection assays. These observations provide a novel notion that vitamin D weakens LPS-induced HIF-1α in a VHL-dependent manner (Fig. 7). The direct mediation of vitamin D/VDR on HIF-1α requires more exploration (Fig. 7).

Our previous works have suggested that oral mucosal VDR signaling protects epithelium against apoptosis by regulating microRNA-802 and p53-upregulated modulator of apoptosis (PUMA) in OLP [30, 35]. In this investigation, we have provided evidence that VDR signaling controls HIF-1α, phospho-p65 and VHL in mouse oral epithelia. We further eliminated bacteria in mouse oral cavity using antibiotics and found that VDR signaling fails to regulate HIF-1α expression without bacteria stimulation, suggesting the indispensable role of LPS in HIF-1α induction. Consistently, TLR4 and HIF-1α levels are robustly elevated in human lesion samples of OLP, whereas VDR and VHL expressions are down-regulated. The status of phospho-p65 in lesion epithelium of OLP has been detected in previous studies [35]. Meanwhile, related cytokines such as IFNγ and IL-1β are also induced largely in the diseased biopsies. These in vivo data reveal critical functions of VDR signaling on HIF-1α expression. As there is no well-established animal model for resembling OLP, we can not offer more in vivo data of mice.

## Conclusion

Taken together, we provide a novel insight into the effect of vitamin D/VDR signaling on oral epithelial inflammation and decipher the molecular basis of this regulation. Despite increasing evidence supports vitamin D’s protective roles in inflammatory diseases, more experimental and clinical studies are needed to ensure vitamin D and its analogs are effective for the management of OLP.

## Additional files

Additional file 1: **Figure S1.** LPS induces cytokines expression in HOKs. (A and B) Real-time PCR quantification of HOKs treated with time course-dependent LPS (100 ng/ml) or saline (A) and concentration-dependent LPS or saline (B) as indicated. (C and D) Elisa measurements of HOKs treated with time course-dependent LPS (100 ng/ml) or saline (C) and concentration-dependent LPS or saline (D) as indicated. *P < 0.05, **P < 0.01, ***P < 0.001 vs. corresponding controls; All assays were conducted for three times, *n* = 3. (TIF 634 kb)

Additional file 2: **Figure S2.** The effects of chetomin and IMD-0354 on HIF-1α in HOKs and TREM-1 expression in human biopsies. (A and B) Western blot analyses of HOKs challenged with LPS or saline in the presence or absence of chetomin (A) and IMD-0354 (B), *n* = 3. (C) TREM-1 levels in human samples. *P < 0.05, **P < 0.01 vs. corresponding normal; Normal, healthy control; Non-inflamed, non-diseased tissue of OLP patients; Inflamed, diseased tissues of OLP patients. Normal, *n* = 7; OLP patients: *n* = 28. (TIF 699 kb)

## Abbreviations

HIF-1α: Hypoxia-Inducible Factor-1α
HOK: Human oral keratinocyte
IKKβ: IκB kinase β
IL-1β: Interleukin-1 beta
IL-6: Interleukin 6
LPS: Lipopolysaccharide
OLP: Oral lichen planus
PHD: Prolyl hydroxylases
PUMA: p53-upregulated modulator of apoptosis
TLR4: Toll-like receptor 4
TNFα: Tumor necrosis factor alpha
VDR: Vitamin D receptor
VHL: Von Hippel-Lindau

## References

[1] De Rossi SS, Ciarrocca K (2014). Oral lichen planus and lichenoid mucositis. Dent Clin N Am.

[2] Zhao Z, Han Y, Zhang Z, Li W, Ji X, Liu X (2018). Total glucosides of paeony improves the immunomodulatory capacity of MSCs partially via the miR-124/STAT3 pathway in oral lichen planus. Biomed Pharmacother.

[3] Gorouhi F, Davari P, Fazel N (2014). Cutaneous and mucosal lichen planus: a comprehensive review of clinical subtypes, risk factors, diagnosis, and prognosis. ScientificWorldJournal..

[4] Cheng YS, Gould A, Kurago Z, Fantasia J, Muller S (2016). Diagnosis of oral lichen planus: a position paper of the American Academy of Oral and maxillofacial pathology. Oral Surg Oral Med Oral Pathol Oral Radiol.

[5] Choi YS, Kim Y, Yoon H-J, Baek KJ, Alam J, Park HK (2016). The presence of bacteria within tissue provides insights into the pathogenesis of oral lichen planus. Sci Rep.

[6] Palazon A, Goldrath AW, Nizet V, Johnson RS (2014). HIF transcription factors, inflammation, and immunity. Immunity..

[7] Goggins BJ, Chaney C, Radford-Smith GL, Horvat JC, Keely S (2013). Hypoxia and integrin-mediated epithelial restitution during mucosal inflammation. Front Immunol.

[8] Lin N, Simon MC (2016). Hypoxia-inducible factors: key regulators of myeloid cells during inflammation. J Clin Invest.

[9] Taylor CT, Doherty G, Fallon PG, Cummins EP (2016). Hypoxia-dependent regulation of inflammatory pathways in immune cells. J Clin Invest.

[10] Mills EL, Kelly B, Logan A, Costa ASH, Varma M, Bryant CE (2016). Succinate dehydrogenase supports metabolic repurposing of mitochondria to drive inflammatory macrophages. Cell..

[11] Fan D, Coughlin LA, Neubauer MM, Kim J, Kim MS, Zhan XW (2015). Activation of HIF-1 alpha and LL-37 by commensal bacteria inhibits Candida albicans colonization. Nat Med.

[12] Rius J, Guma M, Schachtrup C, Akassoglou K, Zinkernagel AS, Nizet V (2008). NF-kappa B links innate immunity to the hypoxic response through transcriptional regulation of HIF-1 alpha. Nature..

[13] Corcoran SE, O'Neill LA (2016). HIF1α and metabolic reprogramming in inflammation. J Clin Invest.

[14] Lee JH, Elly C, Park Y, Liu YC (2015). E3 ubiquitin ligase VHL regulates hypoxia-inducible factor-1α to maintain regulatory T cell stability and suppressive capacity. Immunity..

[15] Bouillon R, Carmeliet G, Verlinden L, van Etten E, Verstuyf A, Luderer HF (2008). Vitamin D and human health: lessons from vitamin D receptor null mice. Endocr Rev.

[16] Haussler MR, Whitfield GK, Haussler CA, Hsieh JC, Thompson PD, Selznick SH (1998). The nuclear vitamin D receptor: biological and molecular regulatory properties revealed. J Bone Miner Res.

[17] Lim WC, Hanauer SB, Li YC (2005). Mechanisms of disease: vitamin D and inflammatory bowel disease. Nat Clin Pract Gastroenterol Hepatol.

[18] Liu W, Chen Y, Golan MA, Annunziata ML, Du J, Dougherty U (2013). Intestinal epithelial vitamin D receptor signaling inhibits experimental colitis. J Clin Invest.

[19] Du J, Chen Y, Shi Y, Liu T, Cao Y, Tang Y (2015). 1,25-Dihydroxyvitamin D protects intestinal epithelial barrier by regulating the myosin light chain kinase signaling pathway. Inflamm Bowel Dis.

[20] Chen Y, Zhang J, Ge X, Du J, Deb DK, Li YC (2013). Vitamin D receptor inhibits nuclear factor kappaB activation by interacting with IkappaB kinase beta protein. J Biol Chem.

[21] Sato Y, Miyauchi Y, Yoshida S, Morita M, Kobayashi T, Kanagawa H (2014). The vitamin D analogue ED71 but not 1,25(OH)2D3 targets HIF1α protein in osteoclasts. PLoS One.

[22] Chen Y, Du J, Zhang Z, Liu T, Shi Y, Ge X (2014). MicroRNA-346 mediates tumor necrosis factor alpha-induced downregulation of gut epithelial vitamin D receptor in inflammatory bowel diseases. Inflamm Bowel Dis.

[23] Du J, Li R, Yu F, Yang F, Wang J, Chen Q (2017). Experimental study on 1,25(OH)2D3 amelioration of oral lichen planus through regulating NF-κB signaling pathway. Oral Dis.

[24] Yin H, Zheng L, Liu W, Zhang D, Li W, Yuan L (2017). Rootletin prevents Cep68 from VHL-mediated proteasomal degradation to maintain centrosome cohesion. Biochim Biophys Acta.

[25] Chen WH, Lecaros RLG, Tseng YC, Huang L, Hsu YC (2015). Nanoparticle delivery of HIF1 alpha siRNA combined with photodynamic therapy as a potential treatment strategy for head-and-neck cancer. Cancer Lett.

[26] Li YC, Pirro AE, Amling M, Delling G, Baron R, Bronson R (1997). Targeted ablation of the vitamin D receptor: an animal model of vitamin D-dependent rickets type II with alopecia. Proc Natl Acad Sci U S A.

[27] Su YY, Chen CD, Guo LJ, Du J, Li XY, Liu Y (2018). Ecological balance of oral microbiota is required to maintain oral mesenchymal stem cell homeostasis. Stem Cells.

[28] van der Meij EH, van der Waal I (2003). Lack of clinicopathologic correlation in the diagnosis of oral lichen planus based on the presently available diagnostic criteria and suggestions for modifications. J Oral Pathol Med.

[29] Cheng YS, Rees T, Jordan L, Oxford L, O'Brien J, Chen HS (2011). Salivary endothelin-1 potential for detecting oral cancer in patients with oral lichen planus or oral cancer in remission. Oral Oncol.

[30] Zhao B, Xu N, Li R, Yu F, Zhang F, Yang F (2019). Vitamin D/VDR signaling suppresses microRNA-802-induced apoptosis of keratinocytes in oral lichen planus. FASEB J.

[31] Wang Y, Zhang H, Du G, Wang Y, Cao T, Luo Q (2016). Total glucosides of paeony (TGP) inhibits the production of inflammatory cytokines in oral lichen planus by suppressing the NF-kappaB signaling pathway. Int Immunopharmacol.

[32] Li YC, Bolt MJ, Cao LP, Sitrin MD (2001). Effects of vitamin D receptor inactivation on the expression of calbindins and calcium metabolism. Am J Physiol Endocrinol Metab.

[33] Yuan W, Pan W, Kong J, Zheng W, Szeto FL, Wong KE (2007). 1,25-dihydroxyvitamin d3 suppresses renin gene transcription by blocking the activity of the cyclic AMP response element in the renin gene promoter. J Biol Chem.

[34] Tu C, Wang S, Hu X, Wang W, Dong Y, Xiao S (2016). Lipopolysaccharide induces TREM-1-dependent HIF-1alpha expression in human keratinocyte cell line. Cell Biol Int.

[35] Zhao B, Li R, Yang F, Yu FY, Xu N, Zhang F (2018). LPS-induced vitamin D receptor decrease in oral keratinocytes is associated with oral lichen planus. Sci Rep.

[36] Fathi MS, El Dessouky HF, Breni HA (2013). CD4+CD25+ T regulatory cells and MMP-9 as diagnostic salivary biomarkers in oral lichen planus. Egypt J Immunol.

[37] Wang H, Zhang D, Han Q, Zhao X, Zeng X, Xu Y (2016). Role of distinct CD4(+) T helper subset in pathogenesis of oral lichen planus. J Oral Pathol Med..

[38] Rouabhia M, Ross G, Page N, Chakir J (2002). Interleukin-18 and gamma interferon production by oral epithelial cells in response to exposure to Candida albicans or lipopolysaccharide stimulation. Infect Immun.

[39] Ohno T, Yamamoto G, Hayashi JI, Nishida E, Goto H, Sasaki Y (2017). Angiopoietin-like protein 2 regulates Porphyromonas gingivalis lipopolysaccharide-induced inflammatory response in human gingival epithelial cells. PLoS One.

[40] Polke M, Seiler F, Lepper PM, Kamyschnikow A, Langer F, Monz D (2017). Hypoxia and the hypoxia-regulated transcription factor HIF-1alpha suppress the host defence of airway epithelial cells. Innate Immun.

[41] Taylor CT, Colgan SP (2017). Regulation of immunity and inflammation by hypoxia in immunological niches. Nat Rev Immunol.

[42] Jiang Y, Zheng W, Teegarden D (2010). 1alpha, 25-Dihydroxyvitamin D regulates hypoxia-inducible factor-1alpha in untransformed and Harvey-ras transfected breast epithelial cells. Cancer Lett.

[43] Lee B, Kwon E, Kim Y, Kim JH, Son SW, Lee JK (2015). 1alpha,25-Dihydroxyvitamin D3 upregulates HIF-1 and TREM-1 via mTOR signaling. Immunol Lett.

[44] Ben-Shoshan M, Amir S, Dang DT, Dang LH, Weisman Y, Mabjeesh NJ (2007). 1alpha,25-dihydroxyvitamin D3 (Calcitriol) inhibits hypoxia-inducible factor-1/vascular endothelial growth factor pathway in human cancer cells. Mol Cancer Ther.

[45] Wu N, McDaniel K, Zhou T, Ramos-Lorenzo S, Wu C, Huang L (2018). Knockout of microRNA-21 attenuates alcoholic hepatitis through VHL/NF-κB signaling pathway in hepatic stellate cells. Am J Physiol Gastrointest Liver Physiol.

[46] Wang J, Lu Z, Xu Z, Tian P, Miao H, Pan S (2017). Reduction of hepatic fibrosis by overexpression of von Hippel-Lindau protein in experimental models of chronic liver disease. Sci Rep.

