# Supplementary figures and images for: Vitamin D/VDR signaling inhibits LPS-induced IFNγ and IL-1β in Oral epithelia by regulating hypoxia-inducible factor-1α signaling pathway
Source: Cell Commun Signal. 2019 Feb 27;17:18. doi: 10.1186/s12964-019-0331-9 (PMC6391768; doi:10.1186/s12964-019-0331-9)

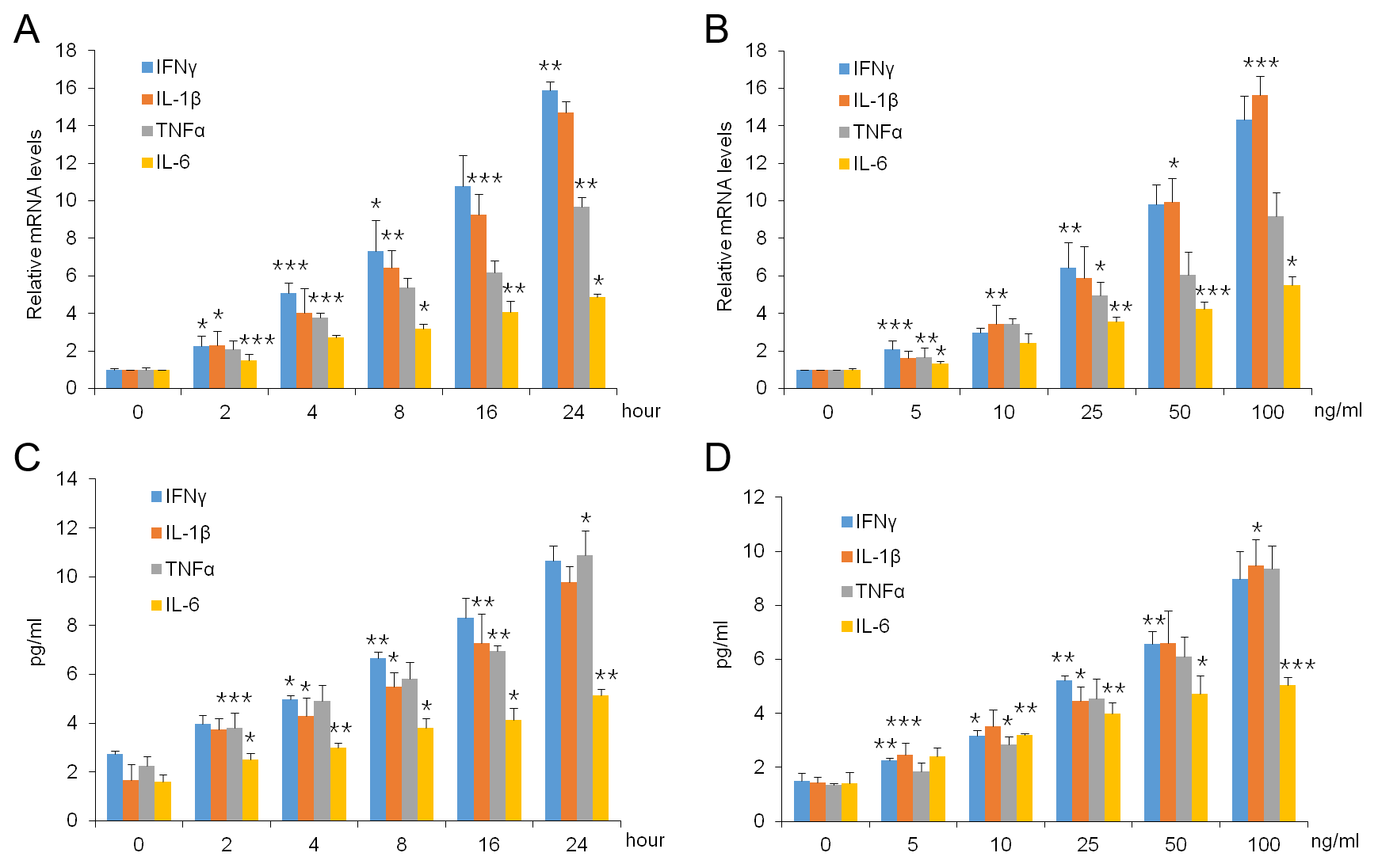

Supplement: Supplementary file 1 — Figure S1. LPS induces cytokines expression in HOKs. (A and B) Real-time PCR quantification of HOKs treated with time course-dependent LPS (100 ng/ml) or saline (A) and concentration-dependent LPS or saline (B) as indicated. (C and D) Elisa measurements of HOKs treated with time course-dependent LPS (100 ng/ml) or saline (C) and concentration-dependent LPS or saline (D) as indicated. *P < 0.05, **P < 0.01, ***P < 0.001 vs. corresponding controls; All assays were conducted for three times, n = 3. (TIF 634 kb) [file 12964_2019_331_MOESM1_ESM.tif]

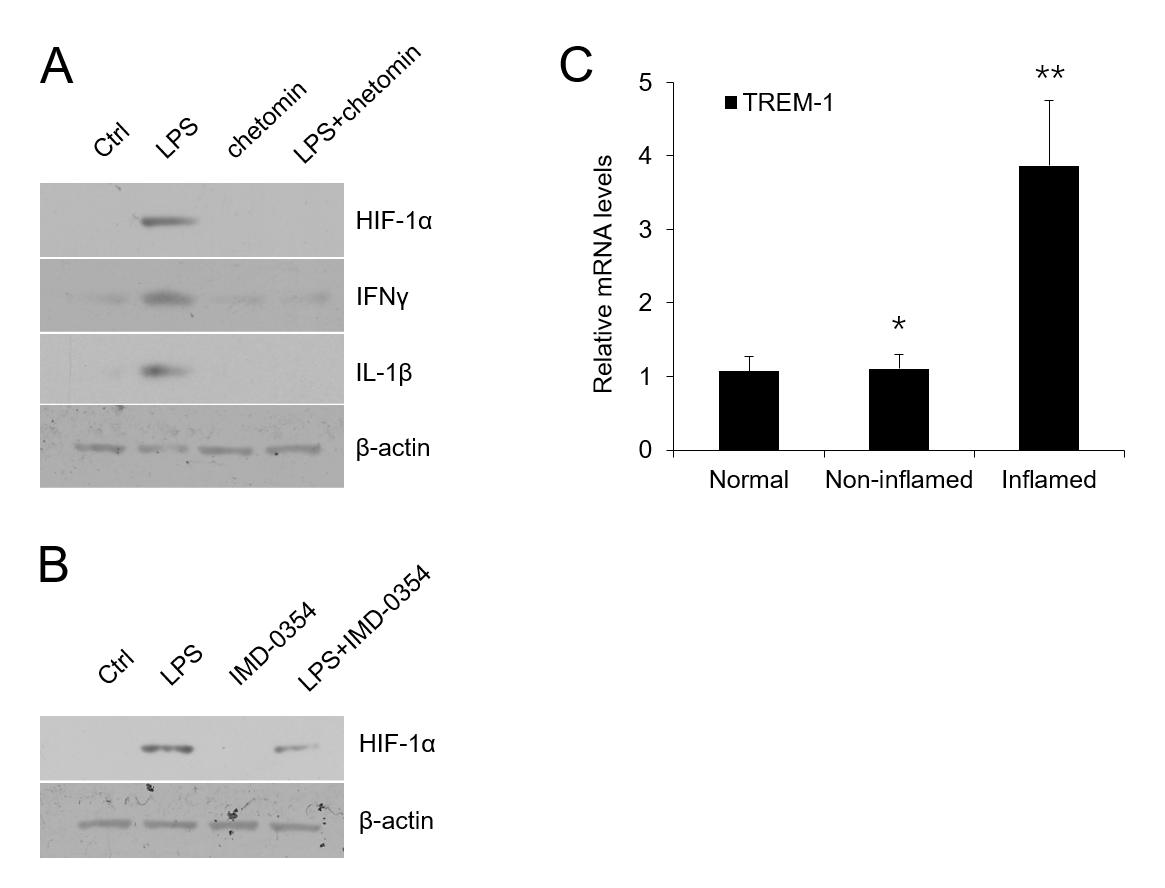

Supplement: Supplementary file 2 — Figure S2. The effects of chetomin and IMD-0354 on HIF-1α in HOKs and TREM-1 expression in human biopsies. (A and B) Western blot analyses of HOKs challenged with LPS or saline in the presence or absence of chetomin (A) and IMD-0354 (B), n = 3. (C) TREM-1 levels in human samples. *P < 0.05, **P < 0.01 vs. corresponding normal; Normal, healthy control; Non-inflamed, non-diseased tissue of OLP patients; Inflamed, diseased tissues of OLP patients. Normal, n = 7; OLP patients: n = 28. (TIF 699 kb) [file 12964_2019_331_MOESM2_ESM.tif]
